# Underdiagnoses of *Rickettsia* in patients hospitalized with acute fever in Indonesia: observational study results

**DOI:** 10.1186/s12879-020-05057-9

**Published:** 2020-05-24

**Authors:** Dewi Lokida, Usman Hadi, Chuen-Yen Lau, Herman Kosasih, C. Jason Liang, Musofa Rusli, Pratiwi Sudarmono, Nurhayati Lukman, Kanti Laras, Rizka Humardewayantie Asdie, Dewi Murniati, I Made Susila Utama, Risna Halim Mubin, Muhammad Karyana, Muhammad Hussein Gasem, Bachti Alisjahbana

**Affiliations:** 1 Tangerang District Hospital, Tangerang, Indonesia; 2 Indonesia Research Partnership on Infectious Disease (INA-RESPOND), Badan Litbangkes, Building 4, 5th floor, Jl Percetakan Negara no 29, Jakarta, 10560 Indonesia; 3 Dr. Soetomo Academic General Hospital – Faculty of Medicine Universitas Airlangga, Surabaya, Indonesia; 4 National Institute of Allergy and Infectious Disease (NIAID), National Institutes of Health, Bethesda, MD USA; 5 Cipto Mangunkusumo Hospital, Jakarta, Indonesia; 6 Sardjito Hospital, Yogyakarta, Indonesia; 7 Sulianti Saroso Hospital, Jakarta, Indonesia; 8 Sanglah General Hospital, Denpasar, Bali Indonesia; 9 Wahidin Sudirohusodo Hospital, Makassar, Indonesia; 10 National Institute of Health Research and Development (NIHRD), Ministry of Health Republic of Indonesia, Jakarta, Indonesia; 11 Dr. Kariadi Hospital - Diponegoro University, Semarang, Indonesia; 12 Hasan Sadikin Hospital – Faculty of Medicine Universitas Padjadjaran, Bandung, Indonesia

**Keywords:** Rickettsioses, Indonesia, Clinical pathway

## Abstract

**Background:**

Reports of human rickettsial infection in Indonesia are limited. This study sought to characterize the epidemiology of human rickettsioses amongst patients hospitalized with fever at 8 tertiary hospitals in Indonesia.

**Methods:**

Acute and convalescent blood from 975 hospitalized non-dengue patients was tested for *Rickettsia* IgM and IgG by ELISA. Specimens from cases with seroconversion or increasing IgM and/or IgG titers were tested for *Rickettsia* IgM and IgG by IFA and *Rickettsia* genomes using primers for *Rickettsia (R.) sp*, *R. typhi*, and *Orientia tsutsugamushi*. Testing was performed retrospectively on stored specimens; results did not inform patient management.

**Results:**

*R. typhi, R. rickettsii*, and *O. tsutsugamushi* IgG antibodies were identified in 269/872 (30.8%), 36/634 (5.7%), and 19/504 (3.8%) of samples, respectively. For the 103/975 (10.6%) non-dengue patients diagnosed with acute rickettsial infection, presenting symptoms included nausea (72%), headache (69%), vomiting (43%), lethargy (33%), anorexia (32%), arthralgia (30%), myalgia (28%), chills (28%), epigastric pain (28%), and rash (17%). No acute rickettsioses cases were suspected during hospitalization. Discharge diagnoses included typhoid fever (44), dengue fever (20), respiratory infections (7), leptospirosis (6), unknown fever (6), sepsis (5), hepatobiliary infections (3), UTI (3), and others (9). Fatalities occurred in 7 (6.8%) patients, mostly with co-morbidities.

**Conclusions:**

Rickettsial infections are consistently misdiagnosed, often as leptospirosis, dengue, or *Salmonella typhi* infection. Clinicians should include rickettsioses in their differential diagnosis of fever to guide empiric management; laboratories should support evaluation for rickettsial etiologies; and public policy should be implemented to reduce burden of disease.

## Background

Rickettsioses are arthropod-borne zoonoses caused by obligate intracellular bacteria from *Rickettsia* or *Orientia* genera. They include murine typhus, spotted fever, and scrub typhus groups [1]. Small mammals serve as reservoirs and arthropods (ticks, fleas, lice, and mites) as vectors. Humans are incidental hosts for many pathogenic rickettsiae [2].

Human rickettsioses in Indonesia are not well characterized. Limited reports have found murine typhus in travelers returning from Indonesia [3–5]. In 2004, over 450 travel-associated cases were reported worldwide; a significant proportion were *R. typhi* from tropical and subtropical areas, *R. conorii* from Southern Asia and *O. tsutsugamushi* from the Asia-Pacific [6, 7]. An active surveillance study of children in Asia showed that 7.6% of Indonesian cases were due to *Rickettsia* [8]. Other fever studies revealed prevalence of murine typhus, spotted fever, and scrub typhus in Northeastern Papua to be 5, 1, and 3%, respectively [9], whereas prevalence of murine typhus in Central Java was 7% [10].

Clinically, rickettsioses are difficult to distinguish from other conditions causing acute fever in endemic areas, especially during the early phase. Common presentations include fever, abdominal discomfort, headache, myalgia, and rashes. Lung, liver, and kidney involvement may complicate the disease [7]. Given the non-specific clinical syndrome and limited access to diagnostics, rickettsioses are likely underdiagnosed in Indonesia. Underdiagnoses could engender inappropriate management, treatment delays, prolonged hospitalisation, and increased morbidity and mortality [11, 12]. Therefore, early diagnosis and empirical therapy of rickettsioses are important.

To characterize the epidemiology of rickettsioses in Indonesia, we performed *Rickettsia* diagnostic panels on blood from subjects in the Acute Fever Requiring Hospitalization (AFIRE) study [13]. Presentation of rickettsial infection in subjects that were initially diagnosed with another infection such as dengue, salmonella and leptospirosis were evaluated to identify features that may confound diagnosis of rickettsiosis.

## Methods

### Study subjects and sample collection

Patients found to have rickettsial infection by reference laboratory testing were identified from INA-RESPOND’s [14] AFIRE observational cohort study conducted in Indonesia from 2013 to 2016. It recruited patients presenting to hospital for evaluation of acute fever, at least 1 year old, hospitalized within the past 24 h, and not hospitalized within the past 3 months. Study sites were eight tertiary hospitals in seven cities in Indonesia: Bandung, Denpasar, Jakarta, Makassar, Semarang, Surabaya and Yogyakarta. Details of AFIRE have been previously described [13].

**Fig. 1:**
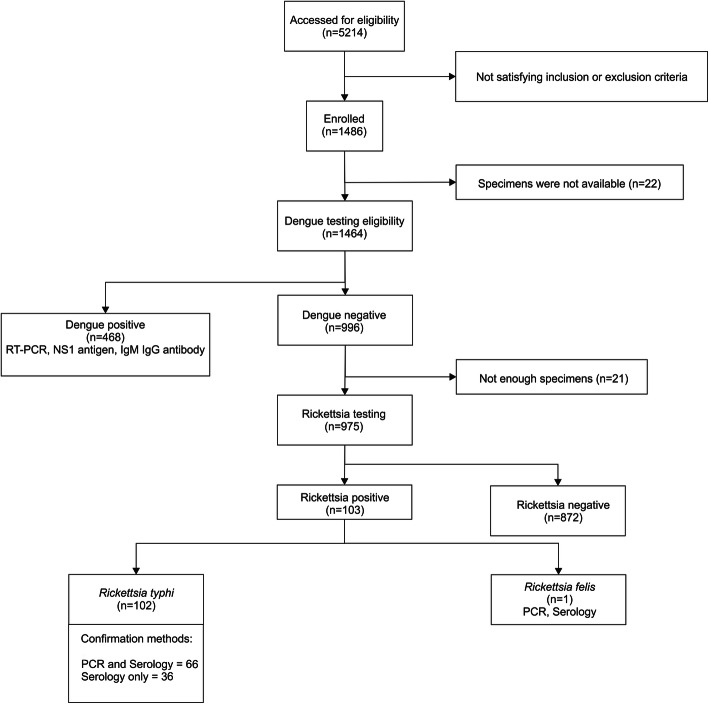
Diagnostic algorithm to identify *Rickettsia* infections

Subjects were evaluated at enrollment, between 14 and 28 days post-enrollment and 3 months post-enrollment. Demographics, clinical data, blood and other clinically indicated specimens were collected during these visits. Blood specimens from the first visit were considered “acute” and specimens from the two follow-up visits were considered “convalescent”. Buffy coat and plasma from blood were stored at − 70 °C and tested retrospectively for pathogens approximately 1 year after enrollment. Specimens from 1464 subjects were first screened for dengue infection. Non-dengue cases were then tested for other pathogens, including *Rickettsia*. Details of the diagnostic procedures are shown in Fig. 1.

### Serologic assay

*R. typhi* IgM, *R. rickettsii* IgM, *R. typhi* IgG, and Spotted fever group IgG were tested using enzyme-linked immunosorbent assay (ELISA) (Fuller Laboratories, San Fransisco, CA). IgM and IgG for Scrub typhus were tested using ELISA (InBios, Seattle, WA). Detailed methods for these assays have been described previously [15, 16]. Convalescent plasma from 975 patients were tested for IgG against *R. typhi*. Due to logistic reasons, only a subset was tested for spotted fever group (*n* = 634) and scrub typhus (*n* = 504). If IgG was positive, IgM and IgG from acute and convalescent plasma were tested simultaneously to assess seroconversion, increase or high optical density and index value in paired samples. In these subjects, indirect immunofluorescence assay (IFA) was performed to detect IgM and IgG reactivity to *R. typhi* and *R. ricketsii* (Focus diagnostics, CA) following the manufacturer’s procedures as previously described [15]. A specimen was considered positive when IgM or IgG fluorescence was observed in the 1:64 dilution. To determine four-fold increase, acute specimens were diluted by two-fold until IgM or IgG fluorescence was not observed. The dilution where IgM or IgG fluorescence was still detected was the end titer of the specimens. The corresponding convalescent specimens was then diluted four-fold of the end titer dilution of the acute specimens. Four-fold increase of IgM or IgG was confirmed when fluorescence was still detected in these diluted convalescent specimens [17]. Sero-conversion of IgM or IgG antibodies was confirmed when no fluorescence was detected in 1:64 dilution in acute specimens but was detected in 1:64 dilution in convalescent specimens.

### Molecular assay

Acute plasma and buffy coat from subjects with sero-conversion or increased IgM/IgG and from subjects that only had acute specimens were tested using PCR. Bacterial DNA was extracted using QIAamp DNA mini kit (Qiagen, Hilden, Germany). *Rickettsia sp.* were detected by the 17-kD outer membrane protein (17-kD *omp*) gene of *Rickettsia sp.*, while *R. typhi* was identified by the *omp*B gene of *R. typhi* following previously published methods [18, 19] and *O. tsutsugamushi* by its 47-kD *omp* gene [20]. Specimens positive for 17-KD *omp* gene of *Rickettsia sp* but not the *omp*B gene of *R. typhi* underwent PCR and nested PCR amplification using primer set RompB3503F, RompB4293R, RompB4246R targeting 743-bp sequence of *Rickettsia sp. omp*B gene and followed by DNA sequencing to determine Rickettsial species [21]. Randomly selected samples with positive *R. typhi* based on qPCR, were confirmed with the same amplification and sequencing method. Sequence chromatograms were edited using BioEdit 7.2.5. software [22]; edited sequences were searched for similarity using BLASTn. Phylogenetic analysis was conducted in MEGA7 and inferred using the Neighbor-Joining method. The analysis involved 704-bp (nucleotide 3556–4259 of *R. typhi* ompB gene).

### Definition for *Rickettsia* diagnosis

*R. typhi* infection was confirmed:

1. When both 17-kD *omp* gene of *Rickettsia* and *omp*B gene of *R. typhi* were detected and/or sero-conversion or four-fold increase of *R. typhi* IgM and/or IgG by IFA was observed.
2. When the detection of 17-kD *omp* gene of *Rickettsia or omp*B gene of *R. typhi* was supported by DNA sequencing of the 743-bp sequence of *Rickettsia sp. omp*B gene or sero-conversion or four-fold increase of *R typhi* IgM and/or IgG by IFA.

Spotted fever group was confirmed by the detection of 17-kD *omp* gene of *Rickettsia* and DNA sequencing of the 743-bp sequence of *Rickettsia sp.omp*B gene and /or sero-conversion or four-fold increase titers of Spotted Fever group IgM and IgG by IFA. Scrub typhus was confirmed by the detection of 47-kD *omp* gene of *O. tsutsugamushi and/or* sero-conversion or four-fold increase *O. tsutsugamushi* IgM and/or IgG by ELISA.

### Statistical analysis

Data were collected in OpenClinica v.3.1 (OpenClinica, LLC) and analyzed using STATA v.15.1 (StataCorp LLC). Clinical and laboratory profiles of confirmed cases were characterized by descriptive statistics and compared according to the three most commonly attributed diagnoses. Proportions were compared between groups using the chi-squared test. The t-test was used to compare means between groups.

### Ethical approval

Ethical approvals were received from IRBs of Dr. Soetomo Hospital (192/Panke.KKE/VIII/2012), Faculty of Medicine Universitas Indonesia Cipto Mangunkusumo Hospital (451/PT02.FK/ETIK/2012) and the National Institute of Health and Research and Development (NIHRD), Ministry of Health, Indonesia (KE.01.05/EC/407/2012).

## Results

Specimens from 975 of 1464 subjects were evaluated using the *Rickettsia* diagnostic panels. *Rickettsia* was identified as the etiology of febrile illness in 103/975 (10.6%) cases (Fig. 1). None of these patients were diagnosed with *Rickettsia* upon clinical presentation. One case was clinically diagnosed as *Rickettsia* during hospitalization but was not laboratory confirmed.

Characteristics of patients with acute rickettsial infection at enrollment are shown in Table 1. Of 103 subjects, 69 (67%) were male and 34 (33%) female. Median age was 35 years (range: 1–75 years), with the majority 53/103 (51.4%) in patients 19 to 45 years. Underlying diseases were documented in 30 subjects, including diabetes (8), hypertension (8), liver disease (3), respiratory diseases (3), anemia (3), malnutrition (3), HIV (1), and osteoarthritis (1).

**Table 1:** Demographics and outcomes (*n* = 103 subjects)

Median age (range) y.o.	35 (1–75)
Male: female	69: 34
Distribution of cases in age group^a^, N(%)	
1 - ≤5 years	2/153 (1.3%)
6 - ≤18 years	19/190 (10%)
19 - ≤45 years	53/378 (14%)
> 45 years	29/254 (11.4%)
Day of onset (median, range), days	5 (1–12)
Length of stay (median, range), days	6 (1–36)
Antibiotics use from 90 subjects with documented history, N (%)	76 (84%)
Ceftriaxone	17 (22.4%)
Ciprofloxacin	9 (11.8%)
Levofloxacin	9 (11.8%)
Cefixime	2 (2.6%)
Cefotaxime, Amoxicillin, Ampicillin, Cefadroxil, Meropenem, Chloramphenicol, Cefoperazone, Sulbactam, Cotrimoxazole	1 (1.3%), each
Combination 2–3 antibiotics	30 (39.5%)
Outcomes, N (%)	
Recovered	72 (69.9%)
Recovered with sequelae^b^	24 (23.3%)
Died	7 (6.8%)
By sites^a^, N (%)	
Bandung	22/189 (11.6%)
Denpasar	16/101 (15.8%)
Jakarta	7/87 (8%)
Makassar	5/131 (3.8%)
Semarang	21/191 (10.9%)
Surabaya	26/156 (16.7%)
Yogyakarta	6/120 (5%)

^a^from 975 subjects tested for *Rickettsia typhi*

^b^Lethargy, arthralgia, anorexia, headache, dizziness, cough

### Confirmation of rickettsial infections

**Fig. 2:**
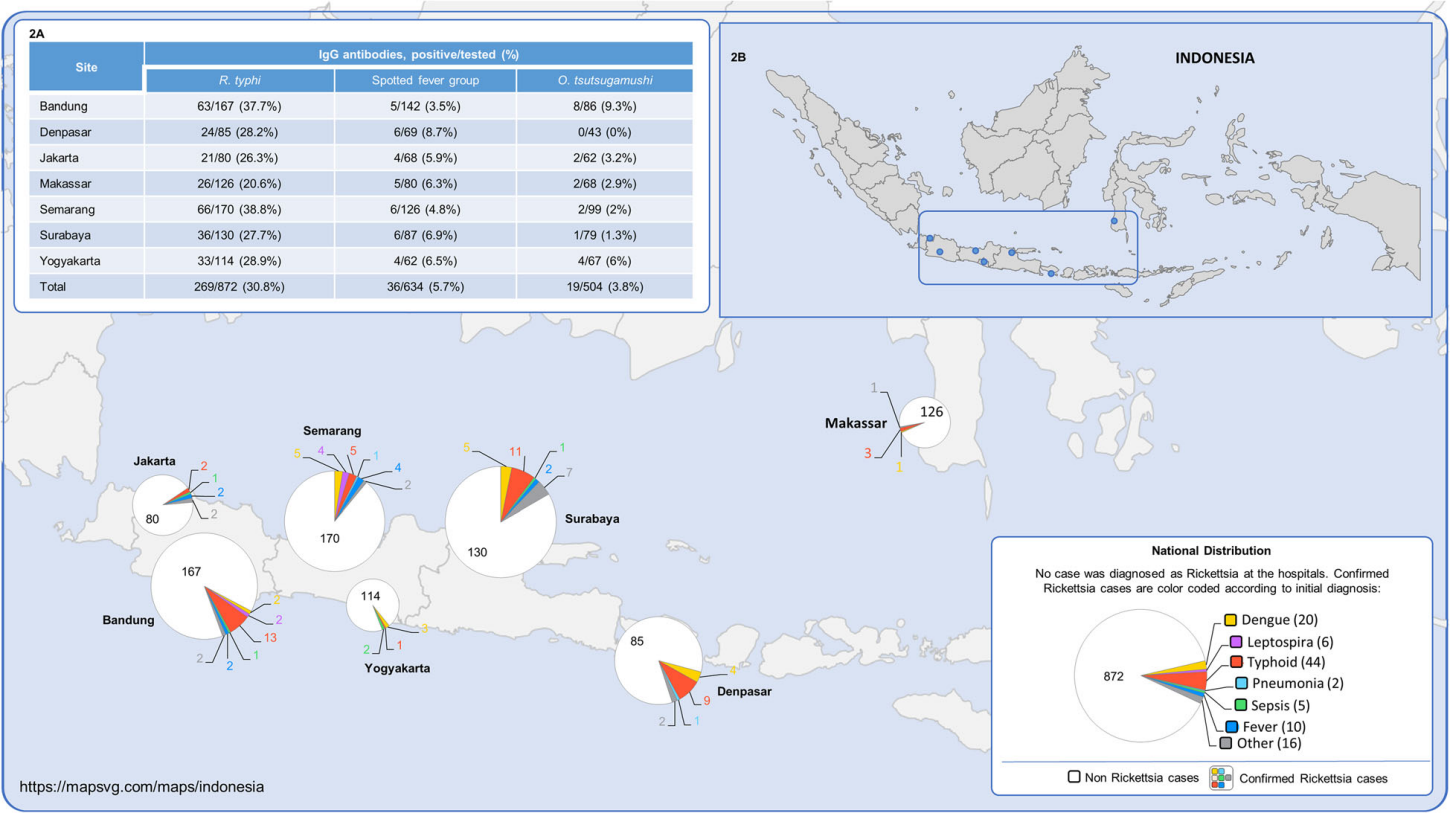
**a** Prevalence of IgG antibodies to *R. typhi*, spotted fever group, and *O. tsutsugamushi* (positive IgG/Subject tested). **b** Geographical distribution of acute *Rickettsia* cases in AFIRE study. Map free source MapSVG. Available from: https://mapsvg.com/maps/indonesia [Accessed 21 March 2020]

The distribution of 103 acute *R typhi* infection cases by site ranged from 5/131 (3.8%) in Makassar to 26/156 (16.7%) in Surabaya. Amongst 872 subjects with no evidence of R typhi infection, *R. typhi* IgG was detected in 269 (30.8%) subjects. Spotted fever IgG and *O. tsutsugamushi* IgG were less common (36/634 (5.7%) and 19/504 (3.8%)), respectively. Geographical distribution of IgG prevalence and acute cases is shown in Fig. 2. In 65 of the 103 subjects with acute *R typhi* infection, confirmation was by *Rickettsia sp* and/or *R. typhi* DNA and sero-conversion or 4-fold increase titers of IgM and/or IgG IFA. In 36 subjects, diagnosis was based on sero-conversion or 4-fold incrase IgM/IgG by IFA. In one subject, *R. typhi* was confirmed by detection of *Rickettsia sp* and sero-conversion of IgM, IgG by IFA. In the one *R. felis* case, increasing IgG titers to *R. typhi* were detected and *R. felis* DNA was identified by *R. felis* PCR using *Rickettsia sp* and *R. felis* primers.

**Fig. 3:**
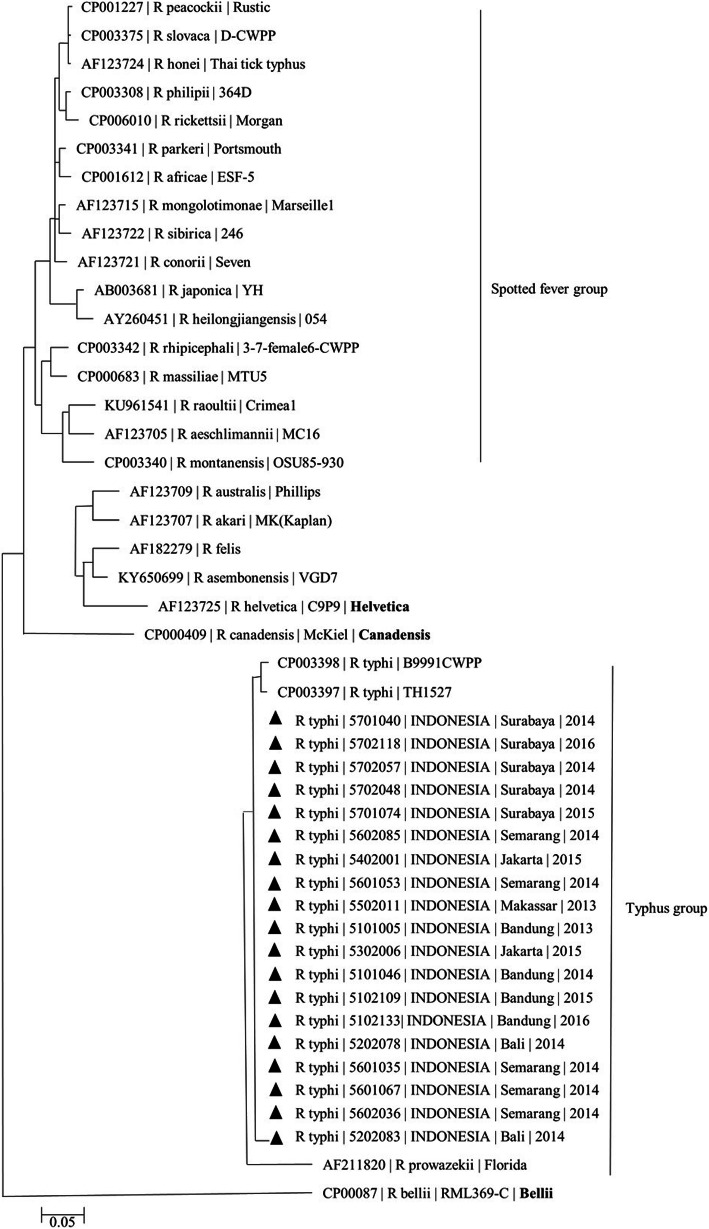
Evolutionary relationships of *omp*B gene *Rickettsia* sp. The evolutionary history was inferred using the Neighbor-Joining method. The analysis involved 704-bp (nucleotide 3556–4259 of *R typhi omp*B gene) of 46 nucleotide sequences. Sequences from subjects of AFIRE study are denoted with black triangle (▲). All positions containing gaps and missing data were eliminated. Evolutionary analyses were conducted in MEGA7

Sequencing of *R. typhi* DNA was conducted in 19 specimens. All revealed 99% sequence identity with *R. typhi* from Myanmar and Thailand (Fig. 3).

### Clinical characteristics

Subjects averaged 5 days (range 1–12 days) of fever before hospital admission (Table 1). Other reported symptoms included nausea (72%), headache (69%), vomiting (43%), lethargy (33%), anorexia (32%), arthralgia (30%), myalgia (28%), chills (28%), and epigastric pain (28%). The clinical triad of *R. typhi* infection (fever, headache and rash) was found in 11%. The three most frequent confirmed diagnoses in the study cohort, dengue, typhoid and leptospirosis demonstrated overlap with rickettsial infection. Details are shown in Table 2.

**Table 2:** Characteristics of *R. typhi* cases, compared to dengue, *S. typhi*, and leptospira cases

Clinical signs and symptoms	*R. typhi* cases	Dengue cases	Salmonella cases	Leptospira cases
	*N* = 102^†^	*N* = 468	*N* = 103	*N* = 48
**Symptoms, N (%)**				
Fever	102 (100)	468 (100)	103 (100)	48 (100)
Nausea	73 (72)	345 (74)	74 (72)	38 (76)
Anorexia	33 (32) ^b^	153 (33)	49 (48)	16 (33)
Headache	70 (69) ^ab^	261 (56)	45 (44)	26 (54)
Vomiting	44 (43)	249 (53)	52 (50)	29 (60)
Lethargy	34 (33) ^a^	102 (22)	46 (45)	14 (29)
Arthralgia	31 (30)	139 (30)	25 (24)	19 (40)
Myalgia	29 (28)	103 (22)	20 (19)	20 (42)
Chills	29 (28) ^a^	65 (14)	35 (34)	20 (42)
Epigastric pain	29 (28)	112 (24)	32 (31)	12 (25)
Cough	24 (24) ^b^	79 (17)	48 (47)	16 (33)
Diarrhea	15 (15) ^bc^	47 (10)	41 (40)	20 (42)
Skin rash	17 (17) ^b^	85 (18)	3 (3)	3 (6)
Constipation	19 (19) ^a^	23 (5)	15 (15)	6 (13)
Altered mental status	4 (4) ^a^	6 (1)	3 (3)	1 (2)
Dysuria	4 (4)	9 (2)	1 (1)	3 (6)
Icterus	5 (5)	2 (0.4)	1 (1)	6 (13)
Rickettsia Triad: fever, headache and rash	11 (11)^b^	57 (12)	2 (2)	1 (2)
**Hematology profiles: Mean (SD)**				
Hb (mg/dl)	14.04 (1.89) ^bc^	13.9 (1.84)	12.4 (1.79)	13.3 (2.06)
Hematocrit (%)	40.8 (5.18) ^b^	41 (5.21)	36.3 (5.42)	38.9 (6.16)
Leukocyte count: /mm^3^	7354 (2975) ^ac^	4225 (2608)	6902 (2974)	10,924 (4246)
Platelet count	123,426 (61,746) ^ab^	107,720 (67,522)	158,402 (74,784)	138,333 (75,491)
**N (%)**				
Leukopenia	14 (14) ^ac^	312 (66.7)	21 (20)	2 (4.2)
Leukocytosis	17 (17) ^a^	9 (1.9)	12 (11.7)	25 (24.5)
Platelet < 100,000/mm^3^	41 (40) ^ab^	266 (57)	22 (21)	13 (27)
Platelet < 150,000/mm^3^	74 (73)	373 (8)	52 (50)	26 (54)
Thrombocytopenia (150,000) AND leukopenia (< 5000)	19 (19) ^ac^	318 (68)	22 (21)	0
**Leucocyte cell types; Mean (SD)**				
Neutrophil (%)	70.34 (10.87) ^abc^	60.59 (16.41)	64.8 (14.2)	84.2 (7.9)
Neutrophil count	5329 (2741) ^abc^	2672 (2227)	4373 (2216)	9001 (3453)
Lymphocyte (%)	21.9 (9.85) ^abc^	28.5 (15.1)	26.5 (12.2)	9.5 (6.4)
Lymphocyte absolute	1388 (484–4489)	888 (192–7104)	1521 (348–10,900)	729 (243–2783)
**N (%)**				
Neutropenia	9 (9)	99 (21)	17 (17)	0
Neutrophilia	12 (12) ^c^	41 (9)	14 (14)	22 (46)
Lymphocytopenia	31 (30)	110 (24)	26 (25)	32 (67)
Lymphocytosis	8 (8)	84 (18)	17 (17)	0
**Biochemistry profiles: Median (range)**				
Bilirubin (mg/dl)	0.55 (0.12–12)	0.37 (0.08–3.46)	0.37 (0.1–2.09)	0.69 (0.13–16.83)
ALT (IU)	82 (12–604)	83 (1–6570)	66 (14–542)	38 (15–207)
AST (IU)	69 (0.71–466)	34 (1–1286)	40 (5–339)	32 (10–141)
Urea (mg/dl)	26 (9–181.2)	17 (0.3–121)	19 (6–59)	53 (13–459)
Creatinine (mg/dl)	0.9 (0.4–9.92)	0.85 (0.05–98)	0.7 (0.3–1.7)	1.2 (0.5–8.8)
**N/available data (%)**				
Bilirubin > 1 mg/dl	21/98 (21) ^abc^	10/409 (2)	5/96 (5)	17/46 (37)
Direct bilirubin > 0.4 mg/dl	36/98 (37) ^abc^	27/409 (7)	13/96 (14)	26/46 (56)
Indirect bilirubin> 0.6 mg/dl	7/98 (7) ^c^	17/409 (4)	2/96 (5)	10/46 (22)
Direct > Indirect bilirubin	66/98 (67)^a^	147/409 (36)	55/97 (57)	34/46 (74)
ALT > 45 IU	76/97 (78)	326/409 (80)	65/95 (68)	16/45 (36)
ALT > 100 IU	35/97 (36) ^c^	147/409 (36)	26/95 (27)	7/45 (16)
AST > 35 IU	75/97 (77)	197/409 (48)	54/97 (56)	16/45 (36)
AST > 100 IU	28/97 (29) ^abc^	60/409 (15)	7/97 (7)	2/45 (4)
Urea *N* > 40 mg/dl	4/31 (13) ^ac^	10/409 (2)	4/44 (9)	24/44 (55)
Creatinine > 1.2 mg/dl	15/98 (15) ^bc^	44/409 (11)	5/94 (5)	25/46 (54)

Note: Significant (*p* < 0.05) ^a^ between *R. typhi* and dengue, ^b^ between *R. typhi* and *S. typhi*, ^c^ between *R. typhi* and leptospira. †One *Rickettsia felis* case is not included

Most subjects presented with normal hematocrit (median 40.8%) and leukocyte count (median 7354/mm^3^). The majority had low lymphocyte proportion (median 21.9%) and platelets (median 123,426/mm^3^). Mildly increased liver enzymes were found in 77%, with bilirubin increases primarily attributable to direct bilirubin. During hospitalization, no clinically relevant changes were observed.

The hematologic profile of *R. typhi* cases was similar to typhoid, but distinguishable from dengue and leptospirosis. Dengue showed lower leukocyte and platelet counts. Leptospirosis showed higher leukocyte and neutrophil counts, but lower absolute lymphocyte counts. Increased total bilirubin and direct bilirubin were more prevalent than in dengue or typhoid, while increased total and indirect bilirubin were more frequent in leptospirosis. AST above 100 IU was more common in *R. typhi* cases compared to the three diseases, whereas creatinine > 1.2 mg/dl was more common in leptospirosis.

### Clinical diagnoses

For the 103 rickettsioses patients, discharge diagnoses were: typhoid fever (44), dengue fever (20), leptospirosis (6), respiratory infections (1 upper, and 6 lower), unidentified fever (6), sepsis (6), hepatobiliary infections (3), unidentified viral infections (3), UTI (3) and others (one each: HIV, chikungunya, enteritis, meningoencephalitis, and diabetic neuropathy).

In all cases of *Rickettsia* initially suspected to be leptospirosis, typhoid fever, chikungunya, or dengue fever, diagnostic assays for those pathogens at the reference laboratory were negative, except in one *R. felis* case where leptospira PCR was positive and leptospira IgM and IgG sero-converted, suggesting co-infection. Clinicians diagnosed typhoid despite negative or weak positive *S. typhi* IgM rapid tests in 24 presumed typhoid cases; in 4 other cases rapid tests were not performed. In the remaining cases [16], positive results from the rapid test were not supported as blood culture, PCR and ELISA IgM tests for *Salmonella* were negative. In these 16 cases, *R. typhi* was confirmed by PCR and/or serological assays. Fifteen dengue diagnoses were not supported by rapid dengue antigen or antibody tests. In contrast, 6 presumed leptospirosis cases had positive rapid tests, but PCR and ELISA at the reference laboratory were negative except in the *R. felis* case above. Details of the diagnostic tests to confirm *Rickettsia* infection and to exclude *S. typhi*, dengue, and *Leptospira* infections are shown in additional file (see Additional file: Table S1).

### Management and outcomes

Antibiotics were taken prior to hospitalization in 23 subjects, including amoxicillin (8), cephadroxil (4), cotrimoxazole (3), chloramphenicol (2), cefixime (1), spiramycin (1), and a combination of antibiotics (4). Antibiotics were given at hospitals in 76 of 90 (84%) subjects with documented treatment data as shown in Table 1. The majority received ceftriaxone (17), ciprofloxacin (9) and levofloxacin (9), or a combination of antibiotics (30). The drug of choice for *Rickettsia* infection, doxycycline was given to 2 patients, one in combination with ceftriaxone and one with amoxicillin. 14 (58%) subjects with suspected viral infections received antibiotics at hospital several days after no clinical improvement with symptomatic treatment. The median hospital stay was 6 days (range 1–36).

Twenty-four subjects (23.3%) recovered with sequelae and 72 (69.9%) recovered without sequelae. Seven (6.8%) patients (median 54.7 years, range 36.1–75 years) died. Of these, 5 had underlying disease (stroke, HIV and chronic liver disease, HIV and TB, DM, and COPD). Six deaths were attributed to sepsis; in one HIV positive patient with meningoencephalitis, death was attributed to cardiogenic shock. All patients who died received antibiotics, however none received doxycycline. Contribution of rickettsial infection to these deaths could not be ascertained.

## Discussion

Our results confirm that rickettsial infections are an important, and often overlooked, cause of fever in hospitalized patients in Indonesia. The prevalence of subjects with *R. typhi* IgG was unexpectedly high (30.8%), suggesting significant population exposure. Furthermore, acute rickettsial infection was the etiology of acute febrile illness in 10.6% of hospitalized subjects, none of whom were clinically diagnosed or managed as having rickettsial infection during hospitalization.

The most common clinical manifestations in our subjects (fever, headache, and nausea/vomiting) have been reported in other studies [23, 24]. However, rash, the hallmark of ricketsial disease diagnosis that usually occurs late (around 5 days of illness in patients with murine typhus) [11] was less common than other reports (17% vs. 12 to 80%, respectively) [24, 25]. This may be attributable to the design of the AFIRE study, which only recorded clinical signs and symptoms of subjects during admission, while other studies monitored them throughout illness. Lack of longitudinal monitoring may explain why prevalence of the *R. typhi* infection clinical triad was lower in our study (11%) than in other studies (49–64%) [26, 27].

Several factors may contribute to the misdiagnosis of rickettsioses during hospitalization. First, presentation of *Rickettsia* infection overlaps with that of other infectious etiologies, particularly typhoid fever, as demonstrated in our study and by data from the US CDC [7]. Second, clinicians may not include rickettsioses in their differential diagnosis. Literature demonstrating the importance of *Rickettsia* infection in the hospital setting is lacking. Previous reports were from a few serologically-confirmed patients from three cities in 1976, 1996 [28], 1997–2000 [9], 2006 [10] and may not have reached clinicians. Third, access to diagnostic tests for rickettsioses is poor and specificity is low for available rapid diagnostics for other pathogens such as *S. typhi*, dengue virus and *Leptospira spp*. Reports from Laos also describe difficulties in differentiating these 4 pathogens [29].

Prior reports also suggest that rickettsioses are an important etiology of fever in Indonesia. A hospital-based study by Groen et al. in Semarang, Central Java found rickettsioses in 7 of 118 (6%) suspected dengue cases during 1995–1996 [28]. A fever study by Gasem et al. in the same city 10 years later reported 20 *Rickettsia* cases amongst 127 children and adults visiting primary health centers and hospitals. It is unclear if these cases were due to *R. typhi* [10]. As part of a dengue vaccine study, Copeding et al. found that 7% of childhood fever was caused by *Rickettsia*, based on ELISA IgM [8].

Previous studies have typically confirmed rickettsioses by serologic assays only. Our study applied a panel of diagnostic assays including molecular assays, ELISA, and IFA, and therefore provides additional information about *Rickettsia* subgroups, as well as the acutely infecting species. Furthermore, this study demonstrated the occurrence of human rickettsiosis in geographical areas excluded from previous studies (Yogyakarta, Surabaya, and Makassar) and reconfirmed that rickettsioses continue to circulate in Semarang, Bandung, Jakarta, and Bali, both in children and adults.

Although the prevalence of rickettsioses in Indonesia was as high as in Thailand or Malaysia, predominant species differed. In our study, *R. typhi* was most common, whereas in Thailand and Malaysia *O. tsutsugamushi* was more frequent [30, 31]. As *O. tsutsugamushi* has been found in hosts and vectors from several areas in Indonesia and evidence of previous infection was detected in our samples, we likely underdiagnosed scrub typhus. This may be because our study did not include primary health centers, where cases of *O. tsutsugamushi* may be managed [9]. It is also possible that there are factors, such as exposure or genetic constitution, in our population that predispose to murine typhus [32].

In 70% of subjects initially diagnosed as typhoid fever or dengue fever, diagnoses were unchanged despite subsequent negative or doubtful rapid test results, suggesting that in the absence of comprehensive diagnostic tests, clinicians had no choice but to judge based on the clinical presentations. In 16 cases with positive (≥4) rapid test, the detection of IgM may be associated with persistent IgM that can be detected more than a year after infection [33] and/or multiple exposure for people living in endemic area [34]. In contrast, the six initially suspected leptospirosis cases had positive *Leptospira* IgM, although reference laboratory confirmatory antibody testing and PCR were negative, except in one case with possible co-infection. This suggests that severe *R. typhi* may clinically resemble leptospirosis (myalgia, abdominal pain, icterus) [29] and clinicians should interpret the results of leptospira antibody tests cautiously. Poor specificity of the Leptospira IgM test, both with rapid testing and ELISA, has been reported [35]. Leukocyte count may also help differentiate between rickettsiosis and leptospirosis as it is more likely to be leukocytosis in leptospirosis.

The case fatality rate of *R. typhi* infection in our study (6.8%) is higher than previously reported (0.3 to 4%) [28–30]. Our study’s higher mortality may be related to presence of comorbidities in 5 of the 7 fatal cases. However, we cannot exclude the possibility of severe *R. typhi*. Complications including meningitis and encephalitis, acute respiratory distress syndrome, acute liver failure, acute renal failure, endocarditis, and multi-organ failure have been reported [36].

Diagnostic inaccuracy can result in inappropriate management of patients. Ideally, rickettsioses should be quickly identified and doxycycline, the antibiotic of choice, administered. Amongst patients with rickettsial infection in the AFIRE study, two subjects initially diagnosed as leptospirosis received doxycycline in combination with other antibiotics. In 58% of suspected viral infection subjects, antibiotics were later given, suggesting that clinicians considered bacterial infections but had difficulty making definitive diagnoses.

Broad spectrum antibiotics such as ceftriaxone or ciprofloxacin are effective against rickettsioses and are reasonable empiric choices while awaiting laboratory confirmation given the overlap of clinical presentations of *Rickettsia* and other infections. However, unnecessary administration of broad spectrum antibiotics should be discouraged. Appropriately targeted treatment could hasten recovery, reduce healthcare utilization, and minimize development of antibiotic resistance [37, 38]. On the other end of the antimicrobial stewardship spectrum, misdiagnosis of rickettsial infection as dengue could result in witholding necessary antibiotics. This is of particular concern in environments where dengue, which is managed supportively, is diagnosed empirically without laboratory confirmation and *Rickettsia* is not considered.

Policy makers and clinicians should prioritize diagnosis, treatment and prevention of rickettsioses in Indonesia. Improved detection with subsequent appropriate management could decrease patient morbidity and reduce healthcare costs. Ideally, laboratories should be equipped with valid diagnostic assays (PCR for molecular detection during acute illness and IFA as the gold standard for serology). However, PCR and IFA have several disadvantages, including the need for an expensive thermal cycler or a fluorescence microscope, which are often unavailable in endemic resource-limited settings, and experienced technicians [39]. Therefore, ELISA can be an alternative when both are unavailable [40]. Proper empiric management, including administration of appropriate antibiotics, and early diagnostic strategies will minimize disease sequelae. Finally, development and implementation of prevention guidelines may also reduce disease burden.

Our study had several limitations. First, we only enrolled hospitalized patients with fever, and therefore the results cannot be generalized to cases not requiring hospitalization. Second, this study was conducted in 7 large Indonesian cities, so may not reflect what is seen in more rural areas or other cities. Finally, as the parent AFIRE study was not designed as a *Rickettsia* study, we did not specifically collect clinical data or request laboratory tests targeting rickettsial infections. To address this limitation, we retrospectively reviewed medical records for additional data not recorded in case report forms. Lastly, we do not know that outcomes would be different if cases had been diagnosed and appropriate targeted treatment provided.

## Conclusions

In conclusion, our study demonstrates the importance of including rickettsioses in the differential diagnosis for fever in hospitalized patients, developing laboratory capacity and point of care test to rapidly and accurately diagnose rickettsioses, and implementing public policy to reduce disease burden. Further studies should be conducted to better characterize the epidemiology of rickettsioses in Indonesia and evaluate outcomes when appropriate empiric and targeted therapy are provided.

## Supplementary information

**Additional file 1**: **Table S1.** Diagnostic tests to confirm rickettsia infection and to exclude *S.* typhi, Dengue, Leptospira, and Chikungunya infections and diagnostic tests to confirm rickettsia infection in an HIV patient and 31 patients with non-rickettsial clinical diagnoses.

## Data Availability

The datasets used and/or analysed during the current study are available from the corresponding author on reasonable request.

## Abbreviations

AFIRE: The etiology of acute febrile illness requiring hospitalization
AST: Aspartate transaminase
ALT: Alanine transaminase
BLAST: Basic local alignment search tool
CDC: Centers for disease control and prevention
COPD: Chronic obstructive pulmonary disease
DNA: Deoxyribonucleic acid
DM: Diabetes mellitus
ELISA: enzyme-linked immunosorbent assay
HIV: Human immunodeficiency virus
IFA: Immunofluorescence assay
INA-RESPOND: Indonesia research partnership on infectious diseases
IU: International unit
PCR: Polymerase chain reaction
TB: Tuberculosis
UTI: Urinary tract infection
