# Supplementary material for: Underdiagnoses of Rickettsia in patients hospitalized with acute fever in Indonesia: observational study results
Source: BMC Infect Dis. 2020 May 24;20:364. doi: 10.1186/s12879-020-05057-9 (PMC7245627; doi:10.1186/s12879-020-05057-9)
Supplement: Supplementary file 1 — Additional file 1: Table S1. Diagnostic tests to confirm rickettsia infection and to exclude S. typhi, Dengue, Leptospira, and Chikungunya infections and diagnostic tests to confirm rickettsia infection in an HIV patient and 31 patients with non-rickettsial clinical diagnoses. [file 12879_2020_5057_MOESM1_ESM.docx]

Supplement Table 1. Diagnostic tests to confirm rickettsia infection and to exclude *S.* typhi, Dengue, Leptospira, and Chikungunya infections and diagnostic tests to confirm rickettsia infection in an HIV patient and 31 patients with non-rickettsial clinical diagnoses.

| **Presumed etiologies OR Clinical Diagnosis / Testing method at sites** | **Rickettsia Confirmation methods at Reference Lab** | |
| --- | --- | --- |
|  | **PCR&/IFA** | **IFA** |
| **Presumed etiologies** | | |
| ***S.* typhi (N=44)^1^** |  |  |
| RDT Salmonella IgM Pos | 11 | 5 |
| RDT Salmonella IgM Neg | 14 | 10 |
| Not done | 2 | 2 |
| **Dengue (N=20)^2^** |  |  |
| RDT Dengue IgM Pos | 3 | 1 |
| RDT Dengue IgM Neg | 9 | 6 |
| Not done | 0 | 1 |
| **Leptospira (N=6)^3^** |  |  |
| RDT/ELISA Leptospira IgM Pos | 4 | 2 |
| **Chikungunya (N=1)^4^** |  |  |
| RDT Chikungunya IgM Pos | 0 | 1 |
| **Clinical Diagnosis** |  |  |
| **Chronic HIV (N=1)** |  |  |
| Not done | 1 | 0 |
| **Respiratory infections (N=7)** |  |  |
| RDT Salmonella/Dengue IgM Neg | 5 | 1 |
| Not done | 0 | 1 |
| **Unidentified Fever (N=6)** |  |  |
| RDT Salmonella/Dengue IgM Neg | 4 | 1 |
| Not done | 0 | 1 |
| **Sepsis (N=6)** |  |  |
| RDT Salmonella/Dengue IgM Neg | 3 | 0 |
| Not done | 2 | 1 |
| **Hepatobiliary infections (N=3)** |  |  |
| RDT Salmonella/Dengue IgM Neg | 1 | 0 |
| Not done | 2 | 0 |
| **Viral infections (N=3)** |  |  |
| RDT Salmonella/Dengue IgM Neg | 3 | 0 |
| **UTI (N=3)** |  |  |
| RDT Salmonella/Dengue IgM Neg | 0 | 3 |
| **Enteritis (N=1)** |  |  |
| RDT Salmonella/Dengue IgM Neg | 1 | 0 |
| **Meningoencephalitis (N=1)** |  |  |
| Not done | 1 | 0 |
| **Diabetes neuropathy (N=1)** |  |  |
| Not done | 1 | 0 |

Lab results at reference lab:  ^1^ Neg blood culture, neg PCR *S.* typhi and *S. paratyphi*, neg IgM *S.* typhi, neg/no increase *S.* typhi IgG ; ^2^ Neg rt-PCR dengue, neg NS1 dengue, neg IgM dengue, no increase IgG dengue ; ^3^ Neg PCR leptospira, neg IgM leptospira, no increase IgG leptospira ; ^4^ Neg rt-PCR chikungunya, neg IgM chikungunya, neg IgG chikungunya
